# Bi- and Tri-specific antibodies in non-Hodgkin lymphoma: current data and perspectives

**DOI:** 10.1038/s41408-024-00989-w

**Published:** 2024-01-25

**Authors:** Iman Abou Dalle, Remy Dulery, Nour Moukalled, Laure Ricard, Nicolas Stocker, Jean El-Cheikh, Mohamad Mohty, Ali Bazarbachi

**Affiliations:** 1 Hematology-Oncology Division, American University of Beirut Medical Center, Beirut, Lebanon; 2 Sorbonne University, Department of Clinical Hematology and Cellular Therapy, Saint-Antoine Hospital, AP-HP, INSERM UMRs 938, Paris, France

**Keywords:** B-cell lymphoma, Cancer immunotherapy

## Abstract

Bispecific antibodies (BsAbs) are a new group of targeted therapies that are revolutionizing the treatment landscape of B-cell non-Hodgkin’s lymphoma (B-NHL). In the relapsed/refractory setting, salvage chemotherapy and autologous stem cell transplantation are capable of curing 50% of patients, whereas the other half will have a dismal outcome with a median overall survival of less than 12 months. This unmet need reinforced the importance of innovative therapies like the BsAbs and CAR-T cell therapies. In this review, we delve into BsAbs in B-NHL from the preclinical development to clinical data in both refractory and frontline settings, and then discuss future perspectives.

## Introduction

B-cell non-Hodgkin lymphomas (B-NHL) are a heterogeneous group of neoplasms comprising over 40 subtypes. The most common types include aggressive diffuse large B-cell lymphoma (DLBCL), and indolent follicular lymphoma (FL) [1]. In most patients, these different B-cell malignancies are curable and/or highly treatable with conventional chemotherapy combined with anti-CD20 monoclonal antibodies (mAbs), an immunotherapy, which has revolutionized the treatment of NHL in the past two decades [2]. However, about 30% of patients develop relapsed and/or refractory (R/R) disease leading to generally poor prognosis [3]. Only 50% of R/R patients are eligible for salvage therapy involving autologous stem cell transplantation (ASCT), and the relapse incidence after ASCT ranged from 35–50% [4]. These patients experience a very short median overall survival (OS) of 10 months, and are in need of innovative therapies that improve the overall response rate (ORR) and, ultimately, the OS. Recently, autologous chimeric antigen receptor (CAR) T- cell therapy has been approved for patients with R/R DLBCL after two prior lines of therapy based on single-arm phase II trials, and as second-line treatment within one year of relapse based on ZUMA-7 and TRANSFORM trials [5–8]. However, the large adoption of CAR T-cell therapy encounters many challenges, such as manufacturing delay, resource availability, and treatment-related toxicities. The emergence of “off-the-shelf” bispecific antibodies (BsAbs) in NHL is an exciting development positioning itself within the therapeutic landscape of NHL. In this review, we delve into BsAbs in B-NHL from the preclinical development to clinical data, both in R/R and frontline settings, and then discuss future perspectives.

## Unmet need in relapsed and/or refractory B-cell lymphomas

Despite the efficacy of rituximab combined with chemotherapy in DLBCL, 25–30% of patients still present with relapsed disease and 20% of these will be refractory to salvage treatments. Those patients have dismal outcomes as their median OS ranges from 6–7 months as per the SCHOLAR-1 trial, regardless of whether they were primary refractory, relapsing after two lines of therapy, or ≤ 12 months after ASCT [9]. Before the era of CAR T-cell therapy, the 2-year OS ranged from 17 to 24%. In FL, 20% of the patients will progress within 2 years. Disease progression within 24 months of treatment has been associated with poorer subsequent OS (hazard ratio [HR] =3.03; 95% CI, 2.65–3.47; *p* < 0.01) compared with patients who did not have progressive disease [10].

Finally, in mantle cell lymphoma (MCL), 20% of patients have high-risk features, namely a high-risk MCL International Prognostic Index (MIPI) score associated with a median OS of 29 months or high-risk molecular features such as *TP53* mutations, or refractoriness to Bruton’s tyrosine kinase (BTK) inhibitors [11, 12].

## Bispecific antibodies

Bispecific antibodies (BsAbs) are manufactured antibody-based molecules with two different antigen-binding sites. T-cell engagers are BsAbs that bind both the target on tumor cells (CD20/CD19 antigens in B-NHL) and immune effector T-cells (CD3 antigen). These T-cell engagers will bypass the major histocompatibility complex (MHC) leading to T-cell activation and tumor cell killing [13].

The first demonstration of the BsAb concept occurred more than fifty years ago in 1964 and the combination of the different heavy (H) and light (L) chain possibilities led, in 1983, to the hybridoma complex that was feasible in only 12% of patients. Later, in 1985, the first demonstration of T-cell redirection with CD3 and a target on the tumour cell occurred. Many challenges were solved by generating H and L chain complexes using complimentary H chains (the strategy known as “Knobs into Holes”) and common light chains.

In 2009, the BsAb catumaxomab, which binds to CD3-EpCAM (epithelial cell adhesion molecule), was the first one to be approved in the European Union (EU). Blinatumomab, a bispecific T-cell engager (BiTE) antibody against CD19 and CD3 was approved in the EU in 2015 and consists of two single-chain variable fragments (scFvs). Its efficacy in B-cell acute lymphoblastic leukemia paved the way for the development of new BsAbs for different targets. In December 2022, the FDA granted accelerated approval to mosunetuzumab-axgb, a BsAb against CD20 and CD3 for adult patients with R/R FL after two or more lines of systemic therapy. Glofitamab, which also targets CD20 and CD3, was also approved in June 2023 for DLBCL patients who have previously received multiple courses of treatment [14, 15].

The first-in-class BsAbs are recombinant, combining two scFvs without an Fc portion, such as blinatumomab [16]. It is very effective in generating a T-cell effector response against the antigen, however, with a very short half-life of 1.5 to 2 hours (h), due to the absence of the neonatal Fc receptor (FcRn) portion, necessitating a continuous intravenous administration. Furthermore, these smaller BsAbs have no Fc-mediated effector functions (such as antibody-dependent cellular cytotoxicity (ADCC), antibody-dependent cellular phagocytosis, and complement activation). Thus, different antibody-based therapy formats now exist: BiTE, bispecific killer cell engager (BiKE), dual-affinity re-targeting (DART), tandem diabodies (TandAbs), diabodies [17].

The next (second) generation of BsAbs (IgG-like) contains Fc domains and has pharmacokinetic (PK) properties of mAbs and a longer half-life. They can generate a long-lasting antitumour immune response and have a silent Fc region, meaning they avoid the Fc-gamma receptor (FcγR)/CD3 crosslinking to reduce toxicity and ADCC. With genetic engineering, different antibody platform technologies such as Knobs into Holes ®, CrossMab ®, DuoBody ® (controlled fragment antigen-binding [Fab]-arm exchange [cFAE]) have solved the issue of H and L chain mispairing, leading to a much more efficient manufacturing process. Now, there is an even newer generation of BsAbs with a different Fc portion such as IgG4 and IgM and a different number of Fab regions (more antigen binding units) leading to bivalent (with 1:1), trivalent (2:1); tetravalent (2:2) antibodies with variable avidity, stabilization of the tumour/T-cell synapse, and cytotoxic potential.

The most advanced CD20 Ig-like T-cell engagers in B-NHL are mosunetuzumab [18], glofitamab [19], epcoritamab [20], odronextamab [21], and plamotomab [22]. All these compounds have an IgG1 Fc portion except odronextamab, which has an IgG4. Their epitopes are different on the CD20 antigen, identical to rituximab for mosunetuzumab, identical to Obinutuzumab for glofitamab and identical to ofatumumab for epcoritamab, and odronextamab. Most are administered intravenously except epcoritamab, which is delivered subcutaneously.

All are administered using a step-up dosing (SUD) process. Mosunetuzumab and glofitamab have a fixed duration of 17 and 12 cycles, respectively, whereas the other three are currently administered until disease progression (Table 1).

**Table 1:** Different characteristics and schedule of CD20xCD3 bispecific antibody in B-cell lymphomas.

Construct	Format	Technology	Route (Max rec. dose)	Schedule	No. of cycles	Indication	ORR / CR rate % (No. pts)	FDA approval
Mosunetuzumab	IGG1	Knobs-into-Holes (different Fabs)	IV (30 mg)/ SC (45 mg)	SUD: 1 mg C1D1, 2 mg C1D8, 60 mg C1D15, 60 mg C2D1, 30 mg C3D1 + every 21 days	8 cycles if CR – up to 17 cycles if PR or SD	R/R FL ≥ 2 lines of tx	80/60 (90)	Dec 2022
Glofitamab	IGG1	Head-to-tail fusion	IV (30 mg)	Obinutuzumab pretreatment 1000 mg, SUD 2.5 mg C1D8 – 10 mg C1D15 – 30 mg C2D1 every 21 days	Max 12 cycles	R/R DLBCL ≥ 2 lines of tx	56/43 (132)	June 2023
Epcoritamab	IGG1	Controlled Fab-arm exchange	SC (48 mg)	SUD: 0.16 mg C1D1, 0.8 mg C1D8, 48 mg C1D15 once weekly on C1-3, Q 2 weeks C4-9, Q 4 weeks C10+	Until disease progression or toxicity	R/R DLBCL ≥ 2 lines of tx	61/38 (157)	May 2023
Odronextamab	IGG4	Heavy chains with different affinity	IV (320 mg)	0.7 mg split: 0.2 mg C1D1/0.5 mg C1D2, 4 mg split: C1D8/D9, 20 mg split: C1D15/D16, 160 mg C2D1 weekly until C4. 320 mg Q 2 weeks C5+	Until disease progression or toxicity	R/R DLBCL or FL ≥ 2 lines of tx	DLBCL: 49/31 (130) FL: 82/75 (121)	Marketing authorization application EMA
Plamotamab	IGG1	Fab-Fc x scFv-Fc	IV (50 mg)	Cycle 1: 0.8 / 2 / 20 / 35 mg QW Cycle 2: 50 mg QW; Cycle 3 onward: 50 mg Q2W	Until disease progression	Mixed R/R B-NHL	NA	NA
IGM 2323	IGM	IgM + modified J chain	IV (0.5–1000)	Days 1, 8, and 15 of 21-day cycles	Until disease progression	R/R B-NHL	N/A	N/A

*Max rec. dose* Maximal recommended dose, *ORR* Overall response rate, *CR* Complete remission, *No*. Number, *FDA* Food and Drug Administration, *IV* Intravenously, *SC* Subcutaneously, *SUD*: Step-up dosing, *C* Cycle, *D* Day, *PR* Partial response, *SD* Stable disease, *R/R* Relapsed and/or refractory, *DLBCL* Diffuse large B cell lymphoma, *FL* Follicular lymphoma, *NHL* Non-Hodgkin lymphoma, *NA* Not applicable, *tx* Treatment.

## Current clinical data on CD3-CD20 bispecific antibodies in NHL

**Fig. 1:**
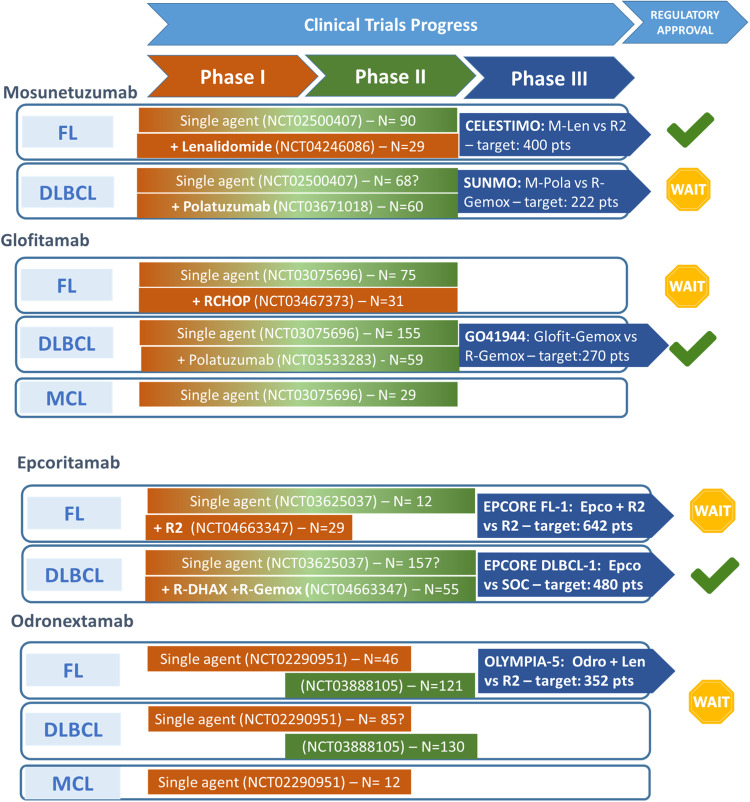
Clinical trial progress of different bispecific antibodies in B-cell non Hodgkin’s lymphomas.

The clinical data on CD20xCD3 BsAbs in B-NHL can be examined by looking firstly at data on efficacy as single-agent therapy, namely mosunetuzumab in FL, mosunetuzumab in elderly DLBCL patients, glofitamab and epcoritamab in DLBCL, and finally glofitamab in MCL (Fig. 1).

### Mosunetuzumab

Mosunetuzumab is a full-length, humanized, immunoglobulin G1-based BsAb targeting CD20 on B-cells and CD3 on T-cells. In a phase II trial, 230 patients with Rr/R B-cell NHL were enrolled and treated at different dose levels [18]. The maximum tolerated dose was not reached (NR). Most common reported adverse events (AE) were neutropenia (28%), cytokine release syndrome (CRS) (27%), hypophosphatemia (23%), fatigue (23%), and diarrhea (22%). Most of them were of low grade, transient, and occurred early in the first cycle. Among 129 evaluable patients with aggressive B-NHL, the ORR was 34.9% and the complete response (CR) rate was 19.4% with a median response duration of 22.8 months in complete responders. Patients with indolent NHL had longer median progression-free survival (PFS) of 11.8 months *versus* 1.4 months for aggressive NHL. The clinical response was strongly associated with the mosunetuzumab exposure.

Mosunetuzumab was one of the first BsAbs developed for R/R FL. A multicentre, single-arm phase II pivotal trial (NCT02500407) [23] included 90 fit adult patients with R/R FL (grade 1–3a), after at least two prior lines of treatment, including an anti-CD20 therapy and an alkylating agent. Mosunetuzumab was administered intravenously in 21-day cycles with SUD for the first cycle to mitigate the risk of CRS: 1 mg on cycle 1 day 1 (C1D1), 2 mg on C1D8, 60 mg on C1D15 and C2D1, and 30 mg on C3D1 and onwards. The treatment continued for 8 cycles in complete responders, and up to 17 cycles in patients with partial response (PR) or stable disease. The patients were heavily pre-treated with a median of three lines of previous therapy (range: 2–10). Twenty-one percent of cases had received an ASCT, 79% were refractory to any prior anti-CD20 therapy, and 52% had disease progression within 2 years from the start of first-line therapy.

After a median follow-up of 18.3 months, a reduction in tumour size was observed in 95% of patients. The ORR and CR rates were 80 and 60%, respectively, and 70% of complete responders maintained response for at least 18 months. The observed CR rate was significantly higher (*p* < 0.0001) than the historical control CR rate of 14% reported with copanlisib therapy, thereby meeting the primary study endpoint. These data led to the regulatory approval of mosunetuzumab for patients with R/R FL after ≥2 prior lines of therapy in 2022.

An updated result of this pivotal phase II study after a median follow-up of 27 months showed a 2-year PFS of 51.4% (95% CI: 39.4–63.3). When compared to the PFS of patients’ last prior therapy (2-year PFS: 23.5%, 95% CI: 14.5–32.5), mosunetuzumab was associated with longer PFS, although limitations should be noted for retrospective comparisons [23]. To put these results into context, they seem comparable to the outcomes of tisagenlecleucel CAR T-cell therapy in the updated ELARA study in patients with R/R FL after 2 lines of therapies that showed a 2-year PFS, duration of response (DOR), and DOR in complete responders of 57%, 65%, and 87%, respectively [24]. Although cross-trial comparisons should be treated with caution, a similar outcome between two innovative therapies is promising.

Mosunetuzumab has a favourable safety profile and can be given in an outpatient setting. In a phase II trial, the most common AE was CRS reported in 40 (44%) of the 90 patients and was predominantly grade 1 with only 2% of grade 3–4 toxicity. CRS events were mostly confined to the first cycle with 23% on D1 and 36% on D15. The median time to CRS was 5 h after D1, 20 h after D8 and 27 h after D15. Fifteen percent of patients with CRS were treated with steroids, 8% received tocilizumab, and 10% needed both. Importantly, 25% of patients were hospitalized for CRS monitoring. The duration of CRS was 3 days. Other most common grade 3–4 AEs were neutropenia (27%) with a median duration of 8 days. There was no febrile neutropenia nor treatment-related fatal AE in this study [25].

A subgroup analysis of this study revealed an interesting finding: patients aged 65 years and above (*n* = 30) had a numerically higher response rate compared to patients younger than 65 years (*n* = 60), with an ORR of 87% *vs*. 77% and a CR rate of 70% *vs*. 55%, respectively. The median DOR was similar (18.7 vs. 22.8 months), and the 18 months event-free survival was 53.5% *vs*. 58.5%, respectively. Both age groups had comparable rates of grade 3–4 AEs (73% *vs*. 68%), mostly attributed to mosunetuzumab. Additionally, older patients had a lower rate of serious AEs of any grade, including CRS that occurred in 30% of older patients compared to 52% in younger patients. Although advancing age is associated with lower immune function, mosunetuzumab showed to be as effective and certainly not more toxic when used in older patients [26].

In R/R aggressive B-cell NHL, mosunetuzumab showed efficacy in the dose escalation, dose-finding trial, with ORR and CR rates of 35% and 19.4%, respectively [18]. A phase I/II multicenter trial investigated mosunetuzumab as a single agent in first-line treatment of elderly unfit DLBCL patients where curative chemo-immunotherapy was precluded due to frailty or reduced organ function [27]. Fifty-four patients were included with a median age of 83 years (range: 65–100). Thirty (56%) had advanced stage (III-IV), and 81% had an International Prognostic Index (IPI) of >2. After a median follow-up of 23 months, ORR and CR rates were 56 and 43%, respectively. Median DOR was 15.8 months (95% CI, 8.5-not estimable [NE]); 11 of 23 responders remained in remission for more than 12 months. CRS and neutropenia were reported in 26 and 15% of patients, respectively, and no neurotoxicity was reported. Mosunetuzumab monotherapy seems to demonstrate promising efficacy with durable responses and a manageable safety profile in this very hard-to-treat population [27]. The combination of mosunetuzumab with a non-cytotoxic agent could be an option worth investigating for this elderly population unfit for chemotherapy.

### Glofitamab

Glofitamab is another potent anti-CD20-directed T-cell engager with a 2:1 molecular format to increase biological efficacy. In vitro, this CD20xCD3 BsAb is capable of inducing a 40-fold tumor lysis capacity by high-avidity bivalent anti-CD20 and head-to-tail orientation of B- and T-cell binding domains as opposed to a classical 1:1 BsAb format. There was also an increase in the synapse formation, an increased binding avidity and stabilization of the tumor-T-cell synapse demonstrated in in-vitro studies. The strong T-cell activation and potent B-cell killing induced by this compound, can definitely lead to greater toxicity from a surge in cytokine release. CRS in this case can be life-threatening and lead to limitations in subsequent dosing. Alternative to the SUD strategy used with mosunetuzumab, a novel approach consists of administering one dose of obinutuzumab 7 days prior to glofitamab dosing, which is sufficient to induce a CD20 + B-cell debulking and abrogate the initial risk of CRS [28].

In a first-in-human phase I trial, glofitamab was administered in dose-escalation steps (0.005–30 mg) to 174 patients with R/R B-cell NHL [19]. These patients were heavily pre-treated with a median of 3 prior therapies, and 90.6% were refractory to their last therapy. Significant clinical activity was observed starting at a dose level of 0.6 mg. At a dose level of 25 mg, almost all patients had CRS, and this was considered as the maximum tolerated D1 dosing. Subsequently, the recommended phase II dose followed the SUD of 2.5 mg (C1D1), 10 mg (C1D8) and 30 mg (C2D1). In all dosed patients, the ORR and CR rates were 53.8% and 36.8% respectively. Among those who received the recommended phase II dose, the responses were higher (ORR: 65.7%, CR: 57.1%). Most responses occurred early in the course of treatment, increasing substantially with dose escalation, with sustained responses in 84% of patients after a maximum observation period of 27.4 months [19]. The most common AE was CRS in 50.3% of all dosed patients, and 71.4% for patients dosed at 2.5/10/30 mg recommended dosing (most were of grade 2: 25.1% and 22.9%, respectively). CRS events were manageable and predictable as most events were confined to the first administration. Other AEs were grade ≥3 neutropenia and infections in 25% and 17.5% of patients, respectively.

The part 2 of this phase I-II study included one hundred and fifty-four patients with R/R DLBCL who had previously received at least two lines of therapy [29]. Patients were heavily pre-treated with a median of three lines of therapy (range: 2–7), including 60% of patients who had received at least three previous therapies and 33% who had previously received CAR T-cell therapy. Patients received an obinutuzumab debulking dose on D1 of the cycle then continued with a SUD based of the recommended phase II dosing 2.5/10/30 mg for up to 12 cycles. After a median follow-up of 12.6 months, 39% patients achieved a CR after a median of 42 days, which coincides with the first response assessment [29]. Responses were seen across subgroups regardless of age, histology, and prior use of CAR T-cell therapy. Notably, while refractory patients presented a lower CR rate (34%) compared to non-refractory patients (70%), the response displayed remarkable durability, with 78% of responders maintaining their response at 12 months. The 1-year PFS and OS were 37 and 50%, respectively [29].

### Epcoritamab

Epcoritamab, another novel CD20xCD30 BsAb, is administered subcutaneously with initial SUD and continued until disease progression. In the dose escalation trial across all B-cell NHL subtypes, escalated doses ranging from 0.0128 mg to 60 mg of single-agent epcoritamab were given to 68 highly refractory CD20+ mature B-cell NHL patients [30]. Dose-limiting toxicities were not identified, and 48 mg was selected as the recommended phase II dose as no additional responses were recorded beyond this dose. Most common AEs were CRS in 59% (all of grade 1 and 2) and injection-site reactions in 47%. Epcoritamab resulted in ORR and CR rates of 68 and 45% in R/R DLBCL and 90 and 50% in R/R FL, respectively [30].

In the dose-expansion cohort of the phase I/II trial, 157 patients with R/R mature B-cell NHL received a median of 5 cycles (15 doses) at the full dose of 48 mg after SUD (0.16 mg on D1 and 0.8 mg on D8) [20]. The ORR and CR rates were 63.1% and 38.9%, respectively. The median time to CR was 2.7 months (range, 1.2–11.1), with an estimated DOR of 12 months. The 6-month PFS rate was 44%; a longer follow-up is needed to determine whether the PFS curve will reach a “plateau”. Epcoritamab demonstrated consistent responses across several pre-specified subgroups, including age, line of therapy, primary refractory disease, and prior exposure to CAR T-cell therapy. The safety profile was as expected from the initial reports of this trial, with the most common AEs being CRS, injection-site reactions and neutropenia [20].

### Bispecific antibodies in mantle cell lymphoma

Despite the recent improvement in the outcome of patients with MCL, there is still an unmet need especially after failure of BTK inhibitors, which will ultimately concern most patients, and those with specific molecular characteristics such as *TP53* mutation or high MIPI score combined with a high Ki67 (prognostic marker) index. Patients with MCL who progress on ibrutinib have a very limited median survival of 2.9 months after ibrutinib discontinuation [11]. Salvage treatments are generally ineffective and lead to responses of short duration [31, 32]. The emergence of CD20xCD3 BsAb for patients with R/R MCL looks encouraging. In a phase II study, 37 patients with R/R MCL were treated with glofitamab SUD after obinutuzumab. Most patients were heavily pre-treated, with 73% considered refractory to their last therapy. The reported ORR and CR rates were 84 and 73%, respectively. Almost all patients had a reduction in tumor volume. The responses were quick and durable as the median time to CR was 51 days and the median DOR was 12.6 months with an estimated 71.6% of patients with CR remaining in response at 9 months. Treatment-related AEs were as expected, CRS in 75.7% and neurologic toxicity in 13.5%; most were of low grade and reversible.

## Mechanism of action of bispecific antibodies

As previously described, BsAbs are T-cell engagers capable of bringing CD3 + T-cells in proximity of the CD20 + B-cells, inducing T-cell activation and subsequent tumor killing. The PK of glofitamab, one of the CD20xCD3 mAbs, were examined in samples from the dose-escalation part of the phase I trial [33]. T-cell margination, activation, and proliferation in the peripheral blood were dose- and response-dependent. The transient reduction in CD8 + T-cells in peripheral blood after a few hours of glofitamab infusion and the magnitude of change was significantly greater in responders compared to other response categories (*p* < 0.002 without adjustment for dose and prognostic factors). In fact, analysed tumor samples demonstrated a spatial reorganization of CD8 + T-cells, characterized by a higher density of cells within the tumor, which resulted in T-cell mediated cell lysis, providing a proof of the underlying mechanism [33].

In addition, the induction of inflammatory cytokines (such as IFN-γ, IL-6, and IL-2) was also dose-dependent, and decreased with subsequent cycles. Interestingly, the cytokine release was not associated with clinical response [33].

## Predictive biomarkers of response and resistance

Taking into consideration the cellular components implicated in the mechanism of action of BsAbs, different resistance pathways may be identified, either related to the tumor cell itself or to the activated T-cells or other non-T cells in the tumor microenvironment (TME). Tumor intrinsic factors (such as TP53, and MYC signalling), and the loss of target antigen (CD20 expression) contribute to tumor immune escape and resistance to BsAb treatment [33–35]. A second mechanism of resistance to BsAbs is intrinsic or acquired T-cell dysfunction within the TME. Although the number of T-cells expressing CD3, and/or CD4 and/or CD8 does not correlate with clinical response, exhausted intra-tumoral T-cells (through high expression of PD-1) is associated with a blunted anti-tumor activity. This resistance may occur owing to the consequences of persistent T-cell receptor triggering, which is known to downregulate CD3 expression and may desensitize intra-tumoral T-cells to further BsAb-dependent activity [36]. Furthermore, continuous stimulation with BsAbs may also induce T-cell exhaustion as opposed to a treatment-free interval [37]. Preclinical data demonstrated improved T-cell functionality, and transcriptional reprogramming after a treatment-free interval [37].

Another potential mechanism of resistance is related to immunosuppressive TME, namely the immunosuppressive myeloid and/or stromal cells, as well as tumor-associated macrophages and cancer-associated fibroblasts [36]. The number of baseline regulatory T cells were shown to be predictive of response in patients treated with blinatumomab for precursor B-cell acute lymphoblastic leukemia [38]. Correlative biomarker studies failed to show an impact of the number of regulatory T cells, natural killer (NK) cells and monocytes on glofitamab response [33, 39]. Further translational research is needed to elaborate on different predictive biomarkers of response and resistance.

## Safety of CD20xCD3 bispecific antibodies

As with all BsAbs, the most frequent AE is CRS. Most cases of CRS are grade 1 or 2, very rarely more severe (grade ≥3). CRS can occur in 15 to 80% of treated patients depending on the trial. With the use of steroids, the CRS rate drops to 0–28%. The second most frequent AE is neutropenia in 15–30% of treated patients, followed by hypophosphatemia (13–29%), anemia (19–38%), fatigue (18–42%) and diarrhea (15–26%) [18, 19].

CRS usually occurs during C1, and its incidence decreases with time [29]. Strategies to mitigate the risk of CRS have been implemented with different methods, such as SUD strategy and pre-treatment with an anti-CD20 mAb. For example, 63% of patients with DLBCL treated with glofitamab experienced CRS, mostly grade 1 (47%) and high-grade CRS was uncommon. The median time to CRS was 13.5 h after the first dose with a median duration of 30 h. There was no reported CRS (grade ≥ 2) in patients after the second and subsequent doses. The use of dexamethasone as pre-medication lowered the incidence from 68 to 48% [29]. For epcoritamab with a slightly different SUD, most CRS events occurred after the first full dose. Most CRS cases were grade 1 and no grade 4 or 5 events were observed. There was slightly more CRS at the third administration at D15 of the C1 and a much lower rate of CRS afterwards [20].

In general, due to prior experience with BsAbs and CAR T-cell therapies, CRS events were substantially reduced, monitored, and treated appropriately. The SUD with 2 or 4 steps depending on the compounds, slow intravenous infusion, mandatory steroid premedication, inpatient administration for the highest dose at risk of CRS, and the pretreatment with obinutuzumab (specific to glofitamab) are mitigating factors that led to a safe administration of the BsAbs. Longer follow-up is needed to ascertain long-term AEs.

## Bispecific antibodies after CAR-T cell therapy failure

Characteristics of both CAR T-cell therapies and BsAbs are summarized in Table 2. Interestingly, BsAbs administration may offer disease control to a substantial portion of patients failing CAR T-cell therapy with CR rates ranging from 24 to 35%. With odronextamab, in patients with DLBCL without previous CAR T-cell therapy who received doses of ≥80 mg, the ORR was 53% (eight of 15) and all responses were CRs. In patients who had previous CAR T-cell therapy and received doses of ≥80 mg, the ORR was 33% (10 of 30) and the CR rate was 27% (8 of 30). Time to response was similar (no prior CAR T-cells: 2.3 months; post CAR T-cells: 1.5 months) as was the DOR (post CAR T-cells: median NR [1.6-NR, longest 29 months]; no CAR T-cells: median NR [2.8-NR, longest 32 months]) [40].

**Table 2:** Comparison of main characteristics of bispecific antibodies versus CAR T-cell therapies.

Characteristics	Bispecific antibody	CAR T-cell therapy
**Structure**	Recombinant protein	Synthetic genetic construct
**T-cell phenotype and effector function**	Endogenous CD8^+^/CD4^+^ cells with higher cytotoxicity	Engineered naïve CD8^+^/CD4^+^ cells
**Activation**	CD3 signal 1 activation Newer generation may use co-stimulation	Signal 1 (CD3-ζ) and signal 2 (CD28, 4-1BB; in 2^nd^ and 3^rd^ generation CAR constructs), and more recently signal 3 (cytokine stimulation ex vivo)
**Immune synapse**	Typical	Atypical
**Availability**	“Off-the-shelf” – immediate use	3 to 5 weeks - In vitro manufacturing
**Manufacturing failures and dosing variability**	Not applicable	Manufacturing failure: < 10% cases Variability in terms of T-cell subset content, CAR transduction efficacy, and number of transfused CAR T-cells.
**Dosing**	Repetitive	Single (after lymphodepleting chemotherapy)
**Administration**	Inpatient and outpatient	Inpatient
**Patient population**	Can be used in elderly unfit patients	Cannot be used in frail and unfit patients
**Cytokine Release Syndrome (Grade 3**+**)**	0–1%	2–23%
**ICANS**	0%	1–28%
**B-cell aplasia and cytopenia**	B-cell aplasia recovers 6–18 months after last infusion Persistent cytopenia: rare	B-cell aplasia months to years after infusion. Cytopenia are common (15–95%) and often persist > 90 days (2–45%).
**Applicability**	Community practice	Specialized centers

*ICANS* Immune effector cell-associated neurotoxicity syndrome.

With epcoritamab, the ORR and CR rates seem to be slightly lower in patients who received CAR T-cell therapy at 54 and 34%, respectively, compared with 69 and 42%, respectively, in those not previously treated with CAR T-cells. The median DOR seems to be comparable with 9.7 months (95% CI, 5.4-NR) for patients with prior CAR T-cells and 12.0 months for CAR T-cell naive patients (95% CI, 5.6-NR) [20, 41]. For glofitamab, 42% of DLBCL post CAR T-cell therapy patients responded and 35% had a CR [42]. Another retrospective series on 9 patients who received glofitamab after CAR T cell therapy failure demonstrated 67% ORR with 4 patients achieving CR [43].

Real-life data showed that for patients with R/R DLBCL who progress after CAR T-cell therapy, salvage treatment with BsAb-based regimens was preferable to standard chemotherapy [44]. The latter study included 217 patients from 12 sites who had a confirmed progression after CAR T-cell therapy. Of these patients, 79 (36%) received palliative care and 138 (64%) received treatment. After the first-line salvage treatment, BsAb-containing treatment led to a notable ORR (CR) of 48% (33%) outperforming immune checkpoint inhibitors 32% (23%) and standard chemotherapy 27% (16%). The only regimen resulting in significant improvement compared to standard chemotherapy was polatuzumab vedotin-piig (PV) treatment, with an ORR (CR) of 62% (38%) and a median PFS at 6.1 months. In contrast, the median PFS was 4.7 months for the BsAb group, 2.7 months for the immune checkpoint inhibitor group, and 2.1 months for the standard chemotherapy group. There may be a PFS plateau of ~25% with BsAbs.

Another multicentric analysis examined late CAR T-cell therapy failure using the DESCAR-T registry (NCT04328298) [45]. It was the first analysis of patients with R/R aggressive B-cell lymphoma who failed (progressive disease or relapse) after 3 months from CAR T-cell therapy. Even though the group numbers were small, the best PFS and OS were observed in the BsAb treatment group. Of 977 patients, 44.1% failed CAR T-cell therapy and of these, 33.6% were late failures (LF). The ORR for CAR T-cell therapy among LF pts was 90.3%, among whom 76 (52.4%) were in complete metabolic response (CMR). After failure, 104 (71.7%) LF patients received a systemic treatment (immunomodulatory drug-based, chemotherapy-based, BsAb, targeted therapy, or anti-PD-1). The ORR after post CAR T-cell treatment failure was 15.4%, with 10.6% in CMR. At a median follow-up from first progression of 15 months, LF patients had a median PFS of 4.2 months (95% CI, 3.4–6) and a median OS of 12.1 months (95% CI, 6.9–15.7) after treatment for CAR T-cell failure. When comparing different therapy groups, in contrast to chemotherapy with a 6-month PFS and OS of 22% and 39.4%, respectively, only patients treated with BsAb showed a substantial benefit in terms of 6-month PFS (77.5%, HR = 0.188 [95% CI, 0.069–0.509], *p* = 0.001). Meanwhile the 6-month OS was longer in both the targeted therapy group (76.6%, HR = 0.278 [95% CI, 0.093–0.83], *p* = 0.02) and BsAb group (92.9%, HR = 0.167 [95% CI, 0.049–0.572], *p* = 0.004). For patients treated with radiotherapy, 6-month PFS and OS were 64.2% (95% CI, 36.9–82.1) and 94.1% (95% CI, 65–99.1), respectively.

## Future perspectives

### Co-stimulation

CAR-induced T-cell activation requires T-cell receptor (TCR) signalling through the CD3 complex (signal 1) and costimulatory CD28 signalling (signal 2). CD8 + T-cells require a third signal, which is cytokine-mediated differentiation and expansion, along with antigen and co-stimulation in order to produce an effective response and avoid death and/or tolerance induction. The lack of co-stimulation (signal 2) after the recognition of antigen (signal 1) has been well demonstrated to induce tolerance and anergy. In T-cell biology, when signal 2 is added with 4-1BB on the T-cell and CD28 on the B-cell, there is T-cell activation, clonal expansion, and effector function. With BsAbs, signal 2 is necessary and the way of adding this is for another BsAb linking 4-1BB and for example, CD19 on the tumor cell.

An ongoing phase I study (NCT04077723) with in vitro data, is examining a costimulatory BsAb (RO7227166) that simultaneously targets CD19 on B-cells and 4-1BB on T-cells in R/R B-NHL patients [46]. The BsAb was initiated after SUD on Cycle 2 D8 and was co-administered with glofitamab (part II of the study) on the same day from C3 onwards. A total of 70 patients (46 with R/R aggressive DLBCL and 24 with R/R indolent B-NHL [including 23 with FL]), with a median age of 66 years, received doses of RO7227166 ranging from 360 mg up to 33 mg. Of 70 safety evaluable patients, 95.7% had adverse events, mostly of grade 1 and 2. Most (83.1%) treatment-emergent AEs (TEAEs) were considered related to glofitamab. No new, additive, or synergistic safety signals were detected, and the overall safety profile was comparable to single-agent glofitamab without an increase in risk of CRS or immune effector cell-associated neurotoxicity syndrome (ICANS) [46]. A PK analysis showed that RO7227166 reversed the expansion of PD-1+ and CD8+ cells with a striking decrease with higher dose administration, which supports the expected mode of action of RO7227166 in combination with glofitamab. Patients with DLBCL achieved a best ORR of 65% and a CR rate of 49%, while the best ORR and CR rate for patients with FL were 91 and 73%, respectively. The median DOR was 109.5 days (*n* = 28). The response was a little disappointing, but at doses explored, combination therapy maintained efficacy similar to single agent glofitamab, and dose escalation continues with additive benefit of the combination expected at higher doses [46].

A trispecific molecule can also provide the second signal (signal 2). The development of a first-in-class anti-CD19, anti-CD3, anti-CD2 IgG-like trispecific antibody (TsAb), PIT565 can also circumvent the issue of T-cell exhaustion. It simultaneously engages CD19+ on the tumor cells, CD3 (TCR signaling component) and CD2 (costimulatory receptor) on T-cells, which leads to redirected T-cell cytotoxicity towards CD19-positive malignant B-cells [47]. In vitro T-cell proliferation, cytokine production, and tumor cell lysis is greater than with BsAbs. There is also more sustained T-cell activity in tumor cell killing and proliferation when compared to BsAbs. The first-in-human trial of PIT565 (NCT05397496) is ongoing, including patients with R/R adult B-NHL after receiving two or more lines of chemotherapy and patients with R/R B-cell acute lymphoblastic leukemia [47].

Other formats of TsAbs can enhance the therapeutic efficacy with co-stimulation. An example from multiple myeloma highlights the ability of a TsAb that interacts with CD38, CD3, and CD28 to enhance both T-cell activation and tumor targeting [48]. The engagement of both CD3 and CD28 affords efficient T-cell stimulation, whereas the anti-CD38 domain directs T-cells to myeloma cells, as well as to certain lymphomas and leukemias. Consequently, TsAbs will have greater T-cell activation and greater in vitro efficacy when compared to BsAbs.

A TsAb can also be used to avoid the immune escape by targeting different antigens, exemplified by Zhao et al., who designed a CD19/CD22/CD3 TsAb [49]. They precisely fused the anti-CD19 scFv (FMC63) and the anti-CD22 nanobody (Nb25) to the defined specific sites on the CD3 antigen-binding fragment (Fab, SP34). This strategy allows for the optimal formation of immune synapses mediated by CD19/CD22/CD3 between target cells and T-cells. As a result, it significantly enhanced antitumor efficacy and effectively overcame immune escape compared to the corresponding BsAbs, whether used alone or in combination, including blinatumomab. Currently, an international phase I first-in-human study (NCT05424822) is ongoing in R/R B-NHL and chronic lymphocytic leukemia patients using anti-CD3-CD20-CD79b (JNJ-80948543).

### Combinations

The compelling evidence for the efficacy and safety of BsAbs in B-NHL led to its addition to the treatment armamentarium of B-NHL. To increase the efficacy of BsAbs, combining them with other known effective agents might be worth exploring, such as lenalidomide, PV, or even cytotoxic chemotherapy.

The combination of lenalidomide and rituximab (i.e., R2) is a well-known combination treatment with high response rates in patients with previously untreated FL [50]. In the updated efficacy and safety results of the RELEVANCE trial at 6 years, R2 continues to demonstrate comparable durable efficacy and safety *versus* chemo-immunotherapy in previously untreated patients with FL and provides an acceptable chemotherapy-free alternative [51]. There is a rationale for combining this effective regimen (R2) with a BsAb to enhance its efficacy through the activation of NK and CD8+ cells.

The data from two phase I/II trials is promising: (1) epcoritamab-R2 compared with R2 (*n* = 30) and (2) mosunetuzumab combined with lenalidomide (*n* = 27) [52, 53]. The median age was 68 years and 59 years, respectively. Both studies included heavily pre-treated R/R FL patients. The rates of patients who progressed within 24 months were 40 and 10%, respectively. Although the follow-up was limited (4 and 5 months, respectively), it appears that the treatment was well tolerated with a CRS rate of 50 and 28%, respectively, with no grade ≥3 in both trials. Neutropenia was present in 47% of patients (24% grade 3–4) and 24% (all grade 3–4), respectively, with an observed incidence lower than with BsAb single agent trials. The ICANS incidence was low at 2 and 3%, respectively. Efficacy was impressive with an ORR (CR) of 95% (80%) after 2 years in the epcoritamab trial and 90% (65.5%) in the mosunetuzumab trial. Based on these promising data, an international phase III trial of epcoritamab in combination with R2 *versus* R2 in patients with R/R FL is presently recruiting (NCT05409066). This is in addition to the arm 6 of the EPCORE NHL-2 trial, which is investigating the safety and efficacy of epcoritamab with R2 as first-line therapy in patients with FL [54, 55]. The latter study included 41 treatment-naive patients with a median age of 57 years, the majority (85%) of whom had stage III or IV disease. The follow-up period was short at 4.4 months. The most common TEAEs included CRS (51%: no grade 3–4), neutropenia (41%: 17% grade 1–2, 24% grade 3–4), pyrexia (41%: all grade 1–2), injection-site reactions (37%: all grade 1–2) and rash (27%: 20% grade 1–2, 7% grade 3–4). Among efficacy-evaluable patients (*n* = 29), the updated ORR was 94% with 86% having a CR. Responses were mostly observed early at first assessment and were durable (median DOR = NR), but a longer follow-up is necessary to assess DOR. There were two fatal TEAEs, both related to COVID-19 and five (12%) treatment discontinuations (three due to an AE and two due to progressive disease) [55]. Mosunetuzumab with lenalidomide as first-line therapy for FL is also under investigation in the United States (NCT04792502).

Another promising BsAb combination is with PV, an antibody-drug conjugate that targets CD79a on tumor cells, linked to a microtubule disruptor (monomethyl auristatin E or MMAE). There are two studies examining PV in combination with a BsAb: (1) a phase Ib/II dose-escalation and dose-expansion study in 63 patients (60 with R/R DLBCL) treated with mosunetuzumab + PV and (2) preliminary data from the use of glofitamab in combination with PV for the treatment of R/R B-NHL (49 patients were evaluable for interim response) [56, 57]. Patients were heavily pretreated in both studies with a median of 3 (range, 1–8) and 2 (range, 1–5) previous lines of therapy, respectively. Median age was 68 years in both studies. In the first study, 24 (40%) patients had been treated with CAR T-cell therapy. The follow-up period was short in both trials (5.7 months and 3.7 months, respectively). In study (1), the CRS rate was 17.5% (grade 1–2; confined to C1) and the rate of ICANS was 8% (3% were grade 3–4). In study (2), most CRS events were grade 1 and occurred after the first dose of glofitamab. There were no reported cases of grade 3 or 4 CRS. The rates of CRS and ICANS were comparable to what was observed with single agents. Neutropenia occurred in 33 and 50%, respectively. As expected, with mosunetuzumab combined with PV, peripheral neuropathy was observed in 42% of patients aged > 65 years, whereas the rate was 21% in younger patients. The incidence of peripheral neuropathy was 17% with the glofitamab and PV combination. There were few AEs leading to treatment discontinuation in both studies.

There was a greater response in elderly R/R DLBCL patients treated with mosunetuzumab and PV (>65 years) with an ORR (CR) of 72% (56%) [56]. The rate for those previously treated with CAR T-cell therapy was 65% (45%). In the glofitamab study, the response rate was high at 80% (51%) [57]. Early durability data are promising, with 97% (28/29) still in CR in study (1) and 92% (23/25) still in CR in study (2) [56, 57].

Epcoritamab is being studied in combination with salvage cytotoxic chemotherapy in the EPCORE NHL-2 trial. This is the first clinical study of a BsAb in high-risk, refractory, ASCT-eligible patients. Updated phase I/II results from arm 4 (epcoritamab + rituximab, dexamethasone, cytarabine, and oxaliplatin or carboplatin [R-DHAX/C]) of this trial included 29 adults with R/R DLBCL who were eligible for ASCT [58]. Patients were treated with standard R-DHAX/C and subcutaneous epcoritamab at 48 mg (21-day cycles: every week in the first 3 cycles). If ASCT was deferred, patients could continue epcoritamab monotherapy until disease progression or unacceptable toxicity. The median age was 58 years (range, 28–75), 72% of patients had received one prior line of therapy, 28% had received ≥ 2 prior lines, and 66% had primary refractory disease. The median duration of follow-up was 9.2 months (range, 1.7–14.2). The incidence of CRS was 41% (grade 1 in 31% of patients, grade 2 in 10% of patients), lower than that observed with single agent epcoritamab. Most events occurred after the first dose, and all were resolved. For the 27 evaluable patients, the ORR was 85% (23/27 patients) and 67% (18 of 27 patients) had a CMR. Median time to response was 1.4 months. After a median follow-up of 12.6 months, 16 patients proceeded to ASCT and all of them remained in continuous remission. Four of the 11 patients who did not proceed to transplant remained on treatment and continued in remission. Patients continuing epcoritamab monotherapy instead of ASCT had an ORR of 64% (7/11), with 45% (5/11) achieving CR. The median DOR, PFS and OS was NR among the seven responders continuing epcoritamab monotherapy [58].

### Combinations in the frontline setting

There are three trials looking at the feasibility of combining a BsAb with standard-of-care chemo-immunotherapy in the frontline setting. The trial of efficacy of mosunetuzumab in combination with CHOP (cyclophosphamide, doxorubicin, vincristine, and prednisone), M-CHOP (mosunetuzumab + CHOP) or CHP-PV in B-NHL is still recruiting (NCT03677141). The efficacy and the safety of the combination in the frontline setting were presented in two studies: in the safety run-in portion and the expansion stage of the ongoing NP40126 study (NCT03467373) using glofitamab with a CHOP regimen [59], and in the updated results of epcoritamab plus rituximab, plus CHOP (R-CHOP) in previously untreated DLBCL and IPI ≥ 3, in arm 1 of the EPCORE NHL-2 study (NCT04663347) [60].

In the glofitamab plus CHOP study, 56 patients were included (46 of whom had reached their scheduled end-of-treatment assessment) with a median age of 68 years (range, 21–84) and median IPI score of 3. The CRS rate was reported in 10.7% of patients, which was much lower than with single-agent glofitamab. After a median follow-up of 5.6 months, the ORR and CR rates were 86% and 76.1%, respectively [59]. In the epcoritamab plus R-CHOP combination study, 33 newly diagnosed DLBCL patients with a median age of 66 years received the combination for 6 cycles, followed by one-year epcoritamab maintenance. All patients had IPI ≥ 3 and ≥24% had double- or triple-hit DLBCL. Median follow-up was 6.9 months and the CRS rate was 51%. In efficacy-evaluable patients, the ORR and CR rates were 100 and 77%, respectively [60]. Longer follow-up is necessary to determine the median DOR and PFS.

### Different structures and Targets

From the perspective of structure and target, the IgM 2323 is a novel T-cell engaging antibody that has been based on an engineered pentameric IgM framework, which gives more physiologic T-cell stimulation and a greater engagement between the T-cell and cancer cell synapse. Initial safety and efficacy data from the phase I dose‐escalation study of IGM‐2323 were presented [61]. This construct has 10 binding domains for CD20 and one single binding domain for CD3. A cohort of 29 patients with R/R B-NHL received this drug at different dose levels. After a median treatment duration of 3.2 (range, 0.0–16.3) months, 58% (23/40) of patients discontinued treatment because of progression. The CRS rate was quite low at 25% (mostly of low grade with only 3% having ≥ grade 3), and the rate of infusion-related reactions was 30% (5% ≥ grade 3). The incidence of neutropenia was also low, observed in 7.5% of patients. The ORR was 29% (11/38) which is somewhat disappointing, especially with fewer than half of the patients with aggressive lymphoma showing a response. Most responders had indolent lymphomas. Phase II randomized dose-selection studies are ongoing: one in DLBCL; the other in FL, with a “pick the winner” strategy using two different doses (Arm A: 15/100 mg and Arm B: 15/300 mg). This will determine the optimal phase II dose in R/R DLBCL and FL, aligning with FDA guidance (Project Optimus).

Another promising BsAb that targets CD19 and CD3 in patients with R/R B-NHL is TNB-486 [62]. Data from a phase I study of TNB-486 showed an ORR of 81.2%, with a CR rate of 68.7% at doses 2.4 mg and higher. For patients with R/R FL, the ORR was 87.5%, and all responders achieved a CR. Responses were also seen among patients with previous CAR T-cell therapy. TNB-486 was engineered with a prolonged half-life of approximately 9–11 days, which would allow for intermittent administration. There were no toxicity issues as compared with other BsAbs.

Instead of T-cells, NK cells, which are part of the innate immune system, constitute an attractive platform for immunotherapy [63]. AFM13 is a first-in-class tetravalent, bispecific NK cell engager that binds to CD30 on tumor cells and CD16A on NK cells. This chimeric construct has two binding sites for each antigen but no Fc domains. By engaging CD16-positive NK cells, AFM13 leads to NK cell-mediated killing of tumor cells [64]. It has very little single-agent activity in classical HL (cHL) probably because NK cells in cHL are largely dysfunctional. In a phase I study, AFM13 was tolerable and demonstrated clinical and pharmacodynamic activity [65]. The ORR was 11.5%, with 3 patients achieving PR and 13 (50%) patients maintaining stable disease, resulting in a disease control rate of 61.5%. To improve the response rates, the authors tried to use cord blood-derived NK cells. The first clinical trial to date to use AFM13 pre-complexed with cytokine-induced memory-like cord blood-derived NK cells that were endowed with a CAR-like specificity in patients with CD30 + R/R HL and NHL was presented [66]. The study included 41 heavily pre-treated CD30-positive HL and NHL patients with a median of seven prior lines of treatment. AFM13 in combination with NK cells showed a very high ORR of 93% and a CR rate of 66%. The CR was 71% at the recommended phase II dose. All four patient who had previously failed CART-cell therapy had a CR. The event-free survival, however, was short in patients not receiving a consolidation with ASCT but may be improved with repeated cycles of this innovative therapy. No instances of CRS or ICANS were observed.

## Concluding remarks

BsAbs stand out as an attractive “off-the-shelf” option for redirecting T-cells against B-cell NHL. Their favourable profiles regarding toxicity and efficacy, along with the possibility of immediate use in most clinical centers, make them one of the most promising drugs in NHL. The first generation of CD20-CD3 BsAbs are already being used in the clinical practice. Perspectives include the use of TsAbs and combining them with NK cells, specialized immune effector cells crucial for activating immune responses against tumor cells. However, many open questions remain unanswered, especially regarding the optimal administration settings, side effects management, pre-treatment strategies, and optimal treatment duration to avoid T-cell exhaustion. Moreover, it is crucial to identify biomarkers of response and gain a deeper understanding of resistance mechanisms. Further research is warranted to determine the optimal sequencing of treatments, including the treatment line (first line *versus* R/R) and the optimal articulation with CAR T-cell therapy (before or after). Finally, identifying which patients could benefit from a re-treatment with BsAbs requires continued investigation.
